# Corneal nerve structure in patients with primary Sjögren’s syndrome in China

**DOI:** 10.1186/s12886-021-01967-7

**Published:** 2021-05-12

**Authors:** Fangting Li, Qin Zhang, Xin Ying, Jing He, Yuebo Jin, Huiwen Xu, Yaobin Cheng, Mingwei Zhao

**Affiliations:** 1 Department of Ophthalmology, Peking University People’s Hospital, Eye Diseases and Optometry Institute, 11 Xizhimen South Street, Beijing, 100044 Xicheng District China; 2 Beijing Key Laboratory of Diagnosis and Therapy of Retinal and Choroid Diseases, College of Optometry, Peking University Health Science Center, Beijing, China; 3 Department of Rheumatology, Peking University People’s Hospital, Beijing, China; 4 Department of Epidemiology and Biostatistics, School of Public Health, Peking University, Beijing, China; 5 Medical Informatics Center, Peking University, Beijing, China

**Keywords:** Corneal nerve, Sjögren’s syndrome, In vivo confocal microscopy (IVCM), Dry eye disease

## Abstract

**Background:**

The aim of this study was to evaluate the in vivo confocal microscopic morphology of corneal subbasal nerves and its relationship with clinical parameters in patients with primary Sjögren’s syndrome in China.

**Methods:**

This was a case control study of 22 dry eye disease (DED) patients with primary Sjögren’s syndrome (pSS) and 20 control subjects with non-Sjögren dry eye disease (NSDE). Each patient underwent an evaluation of ocular surface disease using the tear film break-up time (TBUT), noninvasive tear film break-up time (NIKBUT), noninvasive tear meniscus height (NIKTMH), corneal staining (National Eye Institute scale, NEI), Schirmer I test, meibography, and corneal subbasal nerve analysis with in vivo confocal microscopy (IVCM). The right eye of each subject was included in this study.

**Results:**

SS patients showed a shorter TBUT (*P* = 0.009) and Schirmer I test results (*P* = 0.028) than the NSDE group. However, there was no significant difference in NIKBUT between the two groups (*P* = 0.393). The nerve density of subbasal nerves, number of nerves and tortuosity of the SS group were significantly lower than those of the NSDE group (*P* = 0.001, *P* < 0.001 and *P* = 0.039, respectively). In the SS group, the mean nerve length was correlated with age and the Schirmer I test (r = − 0.519, *P* = 0.013 and r = 0.463, *P* = 0.035, respectively). Corneal staining was correlated with nerve density and the number of nerves (r = − 0.534, P = 0.013 and r = − 0.487, *P* = 0.025, respectively).

**Conclusions:**

Sjögren syndrome dry eye (SSDE) patients have more severe clinical dry eye parameters than non-Sjögren dry eye disease (NSDE) patients. Compared with NSDE patients, we found that SSDE patients showed decreased corneal subbasal nerve density and numbers.

**Supplementary Information:**

The online version contains supplementary material available at 10.1186/s12886-021-01967-7.

## Background

Sjögren’s syndrome (SS) is an autoimmune disorder with external exocrine gland dysfunction and multiorgan involvement [1]. Dry eye is its main clinical manifestation. In primary SS (pSS), the presence of specific antibodies and signs of mononuclear cell infiltration in the exocrine glands accompany reduced tear secretion. These changes can breakdown the homeostasis of the tear film and cause ocular surface inflammation, damage and neurosensory alterations [2–4]. A study has also confirmed that Sjögren syndrome dry eye (SSDE) is not only an aqueous-deficient dry eye but also a mixed type dry eye disease (DED) with both aqueous-deficient dry eye and evaporative dry eye [5].

In dry eye disease, reduced tear secretion leads to inflammation and peripheral nerve damage. Corneal nerves contribute to the reflex control of basal tear production and blinking and regulate corneal epithelial migration and proliferation [6, 7]. Impaired nerve function may evoke a dryness sensation and pain and reduce epithelial recovery. Therefore, the corneal nerve plays a critical role in the pathophysiology of DED [6].

In vivo confocal microscopy (IVCM), as a noninvasive technique, has been widely utilized to evaluate the ocular surface in dry eye disease at the cellular level, including corneal and conjunctival epithelial cell density, conjunctival squamous metaplasia, and corneal nerve morphology [8–11]. In terms of corneal subbasal nerve morphology, controversial results have been demonstrated in several studies among different DED types [12–15]. Previous study investigated the corneal nerve morphology of SSDE concluded that SSDE has more corneal nerve density and number than non-Sjögren dry eye disease (NSDE) in China, which is contrary to studies in western populations [13, 15]. The purpose of this study was therefore to investigate the morphology of corneal subbasal nerves and its relationship with clinical parameters in patients with primary SSDE and NSDE based on a Chinese population.

## Methods

### Study population

This study was conducted at the Peking University People’s Hospital ophthalmology department with the approval of the Medical Ethics Committee of Peking University People’s Hospital. All patients were informed of the aims of the study, and their consent was obtained according to the principles of the Declaration of Helsinki. In this study, data were collected from 22 patients (22 women) with a mean age of 52.36 ± 10.18 years (ranging from 26 to 65 years) affected by SSDE. Patients with pSS were referred to the Peking University People’s Hospital Rheumatology Department. The patients were diagnosed with primary Sjögren’s syndrome according to the American-European Consensus Group (AECG) and American College of Rheumatology (ACR) criteria [16]. A total of 20 patients with DED without Sjögren syndrome (20 women) with a mean age of 38.20 ± 13.37 years (ranging from 25 to 61 years) were recruited as the control group. Non-Sjögren dry eye disease (NSDE) was defined as a tear film break-up time (TBUT) less than 5 s, accompanied by complaints of ocular irritation in the absence of other ocular or systemic diseases [2]. Exclusion criteria for both groups were: aged under 18 years and over 70 years; sarcoidosis, diabetes mellitus, corneal dystrophies and inflammations (infectious keratitis, interstitial keratitis and immunological disorder of cornea (corneal disease in rheumatoid disease, corneal disease with nonrheumatoid collagen-vascular disease, phlyctenular keratoconjunctivitis and Mooren ulcer), systemic therapy with drugs having corneal toxicity, glaucoma, recent use of drugs with anticholinergic properties, use of contact lenses (within 1 month from enrollment) and history of ocular surgery.

### Methods/procedures

All patients underwent a complete examination of the ocular surface of both eyes as follows by a masked operator: tear film break-up time (TBUT), noninvasive tear film break-up time (NIKBUT), noninvasive tear meniscus height (NIKTMH), Schirmer I test, meibography and IVCM analysis of the central cornea subbasal nerves. NIKTMH, NIKBUT and meibography were performed with Oculus Keratograph 5 M (Oculus Keratograph, Oculus, Wetzlar, Germany). Partial or complete loss of meibomian glands was scored using the grades described by Reiko [17]: 0, no loss of meibomian glands; 1, the loss of area was 1/3 of the total meibomian gland area; 2, the loss of area was between 1/3 and 2/3; and 3, the loss of area was more than 2/3. Scores for the upper and lower eyelids were analyzed using ImageJ (ImageJ; National Institutes of Health, Bethesda, MD) and summed to obtain a score for each eye (0–6). TBUT was measured by instilling fluorescein into the inferior cul-de-sac and calculating the average of two consecutive break-up times. Corneal staining was evaluated using the National Eye Institute (NEI) scale after the instillation of fluorescein. Schirmer I test was conducted by inserting Schirmer strips (Jingming Co., Ltd., Tianjing, China) into the lower conjunctival sac at the junction of the lateral and middle thirds without anesthesia for 5 min and wetting of the strips was recorded in millimeters.

### In vivo confocal microscopy

**Fig. 1:**
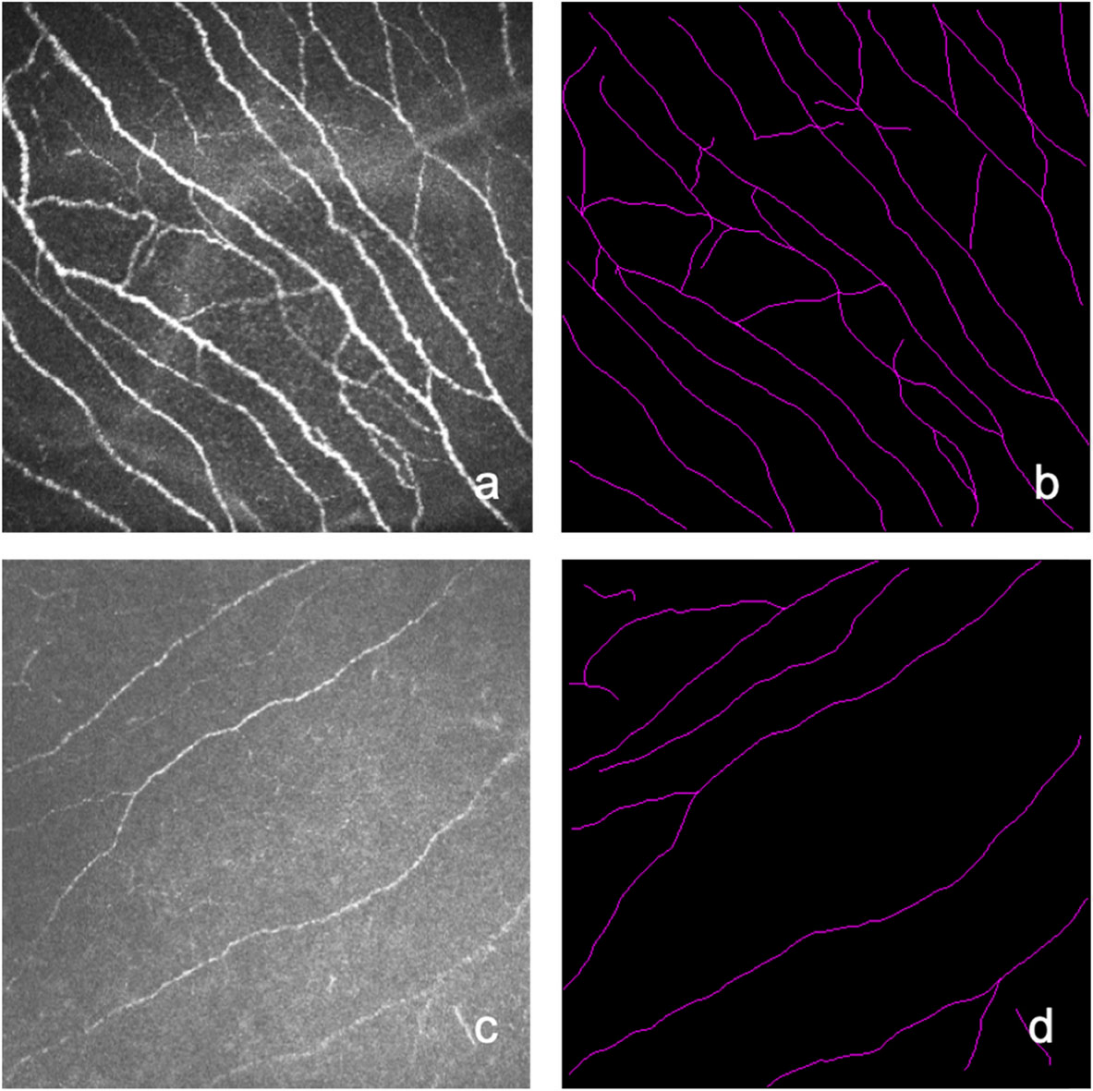
IVCM images of corneal subbasal nerves in the NSDE group **a** and in the SSDE group **c**. Images **b** and **d** are their respective subbasal nerve tracings using NeuronJ software

In vivo laser scanning confocal microscopy was performed using confocal microscopy (Rostock Cornea Module of the Heidelberg Retina Tomograph [HRT/RCM]; Heidelberg Engineering GmbH, Heidelberg, Germany). Images of subbasal nerves of the central cornea were acquired using sequence mode by focusing the microscope beneath the basal epithelium. Approximately 200 corneal subbasal nerve layer images were acquired in the central cornea for each eye. Five images of most subbasal nerve fibers without repeated presence of the same region were selected for quantitative analysis. As described previously, images of corneal subbasal nerves were analyzed retrospectively using NeuronJ (Biomedical Imaging Group, Lausanne, Switzerland) by a single researcher, who was unaware of the patient’s condition and the results of the ocular surface investigations (Fig. 1) [18]. The different parameters used for evaluating the corneal subbasal nerves were: the nerve density (total length of the nerves visible within a frame); the mean nerve length (mean length of nerves and nerve fragments within a frame), maximum length of the corneal nerves (length of the longest nerve or nerve fragment observed within a frame), the number of corneal nerves (sum of the long nerve fiber bundles observed within a frame) and tortuosity (graded 0–4 following the Oliveira-Soto scale) [19]. For each eye and each parameter, the results were the mean of the analysis of five images.

### Statistical analysis

For each patient, the right eye was chosen for statistical analysis. Descriptive statistics were used to analyze the characteristics of the patients using the mean and standard deviation or frequency and percentages. A linear model with heterogeneous variance was used to compare group differences, controlling for age. In addition, to identify relationships between different variables, correlation coefficients (Pearson or Spearman) and age-adjusted partial correlation coefficients (Pearson or Spearman) were calculated. For all tests, the level of significance was set at *P* < 0.05. All analyses were performed using SAS 9.4 (SAS Institute, Inc., Cary, NC).

## Results

There was no difference in terms of sex (*P* = 1.000) between the SS group and NSDE group. The patients with SSDE were significantly older than those in the NSDE group (*P* = 0.001). Concerning ocular surface clinical evaluation, SS patients had a significantly lower TBUT (*P* = 0.009), lower Schirmer I test results (*P* = 0.028) and a higher NEI score (*P* = 0.001) compared with the NSDE group. However, there was no significant difference in NIKBUT between the two groups (*P* = 0.392). SS patients had more meibomian gland dropouts than the NSDE patients, but the difference was not significant (*P* = 0.649). The results of the clinical data are presented in Table 1.

**Table 1:** Demographic and Clinical Test Results

Parameters	NSDE Group	SSDE Group	*P* Value
Patients, *n*	20	22	
Sex			1.000
Female, *n* (%)	20	22	
Male, *n* (%)	0	0	
Age, y	38.20 ± 13.37	52.36 ± 10.82	0.001^*^
NIKTMH, mm	0.16 ± 0.03	0.12 ± 0.05	0.022^*^
NIKBUT, s	4.91 ± 2.04	5.27 ± 2.57	0.392
TBUT, s	4.15 ± 2.01	1.55 ± 1.95	0.009^*^
Corneal Staining (NEI Scale)	1.15 ± 1.50	4.14 ± 3.23	0.001^*^
Schirmer test, mm	10.40 ± 8.29	3.00 ± 4.80	0.028^*^
Meibography	3.10 ± 1.48	3.73 ± 1.28	0.649

^*^ *P* < 0.05

Statistics methods: A linear model with heterogeneous variance was used to compare group differences, controlling for age

In relation to subbasal nerves, the results of the analysis were as follows: SS patients showed significantly lower nerve density (*P* = 0.001). In the comparison of the number of nerves, the SS group was significantly lower than the NSDE group (*P* < 0.001). In addition, greater nerve tortuosity was observed in SSDE (*P* = 0.039). Nevertheless, there were no significant differences in mean length (*P* = 0.057) or maximum length (*P* = 0.229). The IVCM analysis of the subbasal corneal nerves is presented in Table 2.

**Table 2:** IVCM Analysis of Subbasal Nerve Parameters

Parameters	NSDE Group	SSDE Group	*P* Value
Patients, *n*	20	22	
Nerve density μm/frame	3355.71 ± 518.10	2500.10 ± 885.35	0.001^*^
Mean length μm/frame	211.91 ± 30.40	260.94 ± 148.12	0.057
Max length μm/frame	454.19 ± 45.17	443.49 ± 29.04	0.229
Number of nerves number/frame	16.50 ± 3.59	11.63 ± 5.02	0.000^*^
Tortuosity	1.63 ± 1.00	2.42 ± 0.98	0.039^*^

^*^ *P* < 0.05

Statistics methods: A linear model with heterogeneous variance was used to compare group differences, controlling for age

In SS patients, a correlation was found between age and TBUT (r = − 0.647, *P* = 0.001). However, no correlation was found between TBUT and NIKBUT (r = 0.047, *P* = 0.840). NIKBUT was correlated with NIKTMH (r = 0.547, *P* = 0.007). The meibomian gland dropout rate was not correlated with either TBUT or NIKBUT (r = − 0.212, *P* = 0.357 and r = − 0.521, *P* = 0.272, respectively). The results of the correlation between dry eye clinical tests of the SS group are presented in Table 3. Within the NSDE group, we found Schirmer test to be significantly related to NIKBUT (r = − 0.585, *P* = 0.009), TBUT (r = − 0.458, *P* = 0.049) and corneal staining (r = − 0.508, *P* = 0.026). NIKBUT was correlated with TBUT (r = 0.567, *P* = 0.011). No statistical correlations were found between the meibomian gland dropout rate and either TBUT or NIKBUT (r = 0.339, *P* = 0.091 and r = 0.408, *P* = 0.083, respectively). The correlations of the clinical data are shown in Table 4.

**Table 3:** Statistically Significant Correlations Between Dry Eye Clinical Tests in SSDE Group

	Correlation	*P*
NIKBUT - NIKTMH	0.574	0.007
TBUT - age	− 0.647	0.001

Statistics methods: correlation coefficients (Pearson or Spearman) and age-adjusted partial correlation coefficients (Pearson or Spearman) were calculated

**Table 4:** Statistically Significant Correlations Between Dry Eye Clinical Tests in NSDE Group

	Correlation	*P*
NIKBUT - TBUT	0.567	0.011
Corneal staining - age	− 0.698	0.001
Schirmer test - NIKBUT	− 0.585	0.009
Schirmer test - TBUT	− 0.458	0.049
Schimer test - Corneal staining	−0.508	0.026

Statistics methods: correlation coefficients (Pearson or Spearman) and age-adjusted partial correlation coefficients (Pearson or Spearman) were calculated

In the SSDE group, the mean nerve length was correlated with age (r = − 0.534, *P* = 0.013). We found a relationship between mean nerve length and the Schirmer test (r = 0.463, *P* = 0.035). Corneal staining was correlated with both mean nerve length (r = − 0.534, *P* = 0.013) and the number of nerves (r = − 0.487, *P* = 0.025). In the NSDE group, NIKBUT was correlated with nerve density (r = − 0.459, *P* = 0.048), mean nerve length (r = 0.529, *P* = 0.020) and the number of nerves (r = − 0.668, *P* = 0.002). We also observed a significant relationship between the number of nerves and corneal staining (r = − 0.462, *P* = 0.046). The statistical results of the correlation between dry eye and IVCM subbasal parameters in the SS and NSDE groups are presented in Tables 5 and 6, respectively.

**Table 5:** Statistical Results of Correlations Between Dry Eye Clinical Tests and IVCM Subbasal Parameters in SSDE group

Parameters	age	NIKTMH	NIKBUT	TBUT	Corneal Staining	Schirmer test	Meibography
Nerve density							
*r*	0.369	0.197	0.324	0.082	−0.534	− 0.203	0.144
*P*	0.091	0.392	0.152	0.724	0.013^*^	0.378	0.534
Mean length							
*r*	−0.519	−0.103	−0.279	0.116	0.316	0.463	−0.146
*P*	0.013^*^	0.656	0.221	0.616	0.163	0.035^*^	0.527
Max length							
*r*	0.246	0.154	−0.143	−0.009	− 0.069	−0.285	− 0.115
*P*	0.269	0.506	0.536	0.969	0.767	0.211	0.620
Number of nerves							
*r*	0.421	0.208	0.246	0.027	−0.487	−0.268	0.184
*P*	0.051	0.366	0.283	0.908	0.025^*^	0.241	0.424
Tortuosity							
*r*	−0.024	−0.343	0.034	−0.285	0.057	0.109	0.011
*P*	0.917	0.128	0.883	0.211	0.807	0.637	0.963

^*^ *P* < 0.05

Statistics methods: correlation coefficients (Pearson or Spearman) and age-adjusted partial correlation coefficients (Pearson or Spearman) were calculated

**Table 6:** Statistical Results of Correlations Between Dry Eye Clinical Tests and IVCM Subbasal Parameters in NSDE group

Parameters	age	NIKTMH	NIKBUT	TBUT	Corneal staining	Schirmer test	Meibography
Nerve density							
*r*	−0.001	−0.068	− 0.459	0.097	− 0.357	0.136	0.12554
*P*	0.996	0.781	0.048^*^	0.693	0.134	0.579	0.6086
Mean length							
*r*	−0.421	0.270	0.529	0.348	0.331	−0.283	0.23813
*P*	0.065	0.263	0.020^*^	0.144	0.167	0.241	0.3262
Max length							
*r*	0.158	0.181	0.001	0.320	0.079	−0.290	0.33177
*P*	0.506	0.460	0.996	0.182	0.747	0.229	0.1652
Number of nerves							
*r*	0.303	−0.211	−0.668	0.009	−0.462	0.351	−0.07844
*P*	0.194	0.386	0.002^*^	0.972	0.046^*^	0.141	0.7496
Tortuosity							
*r*	0.055	0.302	0.099	0.306	0.195	0.052	−0.02672
*P*	0.817	0.208	0.688	0.202	0.423	0.833	0.9135

^*^ *P* < 0.05

Statistics methods: correlation coefficients (Pearson or Spearman) and age-adjusted partial correlation coefficients (Pearson or Spearman) were calculated

## Discussion

In the present study, our study found that subbasal nerve density was significantly lower in the SSDE group than in the NSDE group. The mean length and maximum number of nerves were not significantly different between the two groups. SSDE patients had fewer nerves than NSDE patients. Therefore, we considered that this apparent difference may be attributed to the smaller numbers of nerves of SSDE but not the shorter length of each nerve. Previous studies focusing on corneal subbasal nerves in patients with SSDE and NSDE have demonstrated controversial results regarding nerve density. Most of studies concluded there was a decrease in nerve density in both SSDE and NSDE [18, 20, 21]. Nevertheless, Tuominen et al. [22] and Hosal et al. [23] reported unchanged nerve density in DED patients. While Zhang et al. reported increased corneal nerve density of SSDE in a Chinese population which is contrary result to ours, and they hypothesized that the increase in nerves was an indication of nerve regeneration [14]. In our opinion, the controversial finding might be caused by either different stages or severity of the disease which might induce different degeneration/regeneration patterns of nerves, levels of inflammation or levels of corneal hyperalgesia and allodynia [6]. Our results showed that SSDE patients suffered decreased corneal nerve density or were in a nerve-damaging stage. Most patients in the SSDE group had severe DED, so decreased corneal nerve density might be a later period of SSDE after nerve regeneration or serious inflammation of the ocular surface of SSDE sabotaged nerve regeneration. Forty percent of primary Sjögren’s syndrome patients experience chronic neuropathic pain, and skin biopsies show either reduced epidermal nerve fiber (ENF) density or abnormal morphology [24–26]. Therefore, the reduction in the corneal subbasal nerves might be primary damage due to pSS or a combined consequence of secondary damage from dry eye disease and pSS.

The two techniques of tear break-up time should be correlated, and the noninvasive technique (NIKBUT) is about 2.0 s longer than TBUT [27, 28]. In our study, NIKBUT of SSDE was not correlated with TBUT with a mean difference of 3.5 s, while NIKBUT had a relationship with TBUT and NIKBUT was 0.7 s longer than TBUT in the NSDE group. A significant drawback of TBUT is that the measurement of TBUT depends on subjective assessment by the observer. The SSDE patients in our study had severe DED. TBUT observer’s assessment may have been interfered with by a worse fluorescein staining status. This might explain the tear break-up time discrepancy of the two techniques. Future studies should investigate the best way to evaluate the tear break-up time of severely dry eye patients, especially among those with Sjögren’s syndrome.

This study found that dry eye clinical parameters of the SSDE group were worse than those of the NSDE group. SSDE patients had shorter TBUTs and lower NIKTMH and Schirmer test results than the NSDE group. Previous studies have shown that SSDE also manifests evaporative dry eye [5] and has a higher frequency of severe meibomian gland dysfunction (MGD) than NSDE [8]. In our study, SSDE patients suffered more meibomian dropouts accompanied by a lower TBUT, suggesting that SSDE might have more severe MGD. The SSDE group had more meibomian gland dropout than the NSDE group, but the difference was not significant. Meanwhile, the meibomian gland dropout rate was not associated with the dry eye clinical parameters in either group. The meibomian gland dropout rate is one of the parameters used for evaluating the meibomian gland, which may not represent meibomian gland function or meibum quality [29]. Therefore, dropout ratio was not associated with corneal staining, NIKBUT or any other parameters in this study. This indicates that even though SSDE is a combined dry eye, the meibomian gland structural variations is not the prime pathogenesis of SSDE.

The major limitation of these studies arises from the inhomogeneous distribution of nerve fibers across the area of the cornea [30]. A single IVCM image (typically 0.16 mm^2^) is insufficient for reliable morphometric characterization of the subbasal nerve plexus. Three to eight multiple nonoverlapping IVCM images are recommended to effectively expand the examined corneal area [31, 32], which have to be manually selected from a larger set of acquired images according to predefined quality criteria. However, several selected IVCM images may not fully represent the subbasal nerve condition of the patient, which will cause systemic error. Various alternative approaches to increase the examined area have been developed, such as the EyeGuidance system [33]. Composite images covering larger areas or even the whole cornea subbasal nerve will certainly provide more reliable evaluations in the future. Another limitation of our study is the relatively small sample size.

## Conclusions

In our study, SSDE patients showed decreased corneal subbasal nerve density and numbers of nerves compared with the NSDE group. We conclude that corneal subbasal nerve decreases in SSDE, this difference may be attributed to the fewer numbers of nerves of SSDE. And decreased corneal nerve may contribute to the pathophysiology of SSDE. Therefore, further corneal nerve investigations could improve our understanding and treatment of SSDE.

## Supplementary Information

**Additional file 1: Supplementary Table 1**. Statistical Results of Correlations Between Dry Eye Clinical Tests in the SSDE Group. **Supplementary Table 2**. Statistical Results of Correlations Between Dry Eye Clinical Tests in the NSDE Group.

## Data Availability

The datasets used and/or analyzed during the current study are available from the corresponding author on reasonable request.

## Abbreviations

IVCM: **I**n vivo confocal microscopy
DED: **D**ry eye disease
pSS: **P**rimary Sjögren’s syndrome
NSDE: **N**on-Sjögren dry eye disease
TBUT: **T**ear film break-up time
NIKBUT: **Noninvasive** tear film break-up time
NIKTMH: **Noninvasive** tear meniscus height
SSDE: Sjögren syndrome dry eye
SS: Sjögren’s syndrome
AECG: American-European Consensus Group
ACR: American College of Rheumatology
NEI: National Eye Institute
MGD: Meibomian gland dysfunction
ENF: Epidermal nerve fiber

## References

[1] Patel R, Shahane A (2014). The epidemiology of Sjögren’s syndrome. Clin Epidemiol.

[2] Craig JP, Nelson JD, Azar DT, Belmonte C, Bron AJ, Chauhan SK, de Paiva CS, Gomes JAP, Hammitt KM, Jones L, Nichols JJ, Nichols KK, Novack GD, Stapleton FJ, Willcox MDP, Wolffsohn JS, Sullivan DA (2017). TFOS DEWS II report executive summary. Ocul Surf.

[3] Solomon A, Dursun D, Liu Z, Xie Y, Macri A, Pflugfelder SC (2001). Pro- and anti-inflammatory forms of interleukin-1 in the tear fluid and conjunctiva of patients with dry-eye disease. Invest Ophthalmol Vis Sci.

[4] Tincani A, Andreoli L, Cavazzana I, Doria A, Favero M, Fenini M (2013). Novel aspects of Sjogren’s syndrome in 2012. BMC Med.

[5] Shimazaki J, Goto E, Ono M, Shimmura S, Tsubota K (1988). Meibomian gland dysfunction in patients with Sjogren syndrome. Ophthalmology..

[6] Belmonte C, Nichols JJ, Cox SM, Brock JA, Begley CG, Bereiter DA, Dartt DA, Galor A, Hamrah P, Ivanusic JJ, Jacobs DS, McNamara NA, Rosenblatt MI, Stapleton F, Wolffsohn JS (2017). TFOS DEWS II pain and sensation report. Ocul Surf..

[7] Garcia-Hirschfeld J, Lopez-Briones LG, Belmonte C (1994). Neurotrophic influences on corneal epithelial cells. Exp Eye Res.

[8] Erdelyi B, Kraak R, Zhivov A, Guthoff R, Nemeth J (2007). In vivo confocal laser scanning microscopy of the cornea in dry eye. Graefes Arch Clin Exp Ophthalmol.

[9] Villani E, Magnani F, Viola F, Santaniello A, Scorza R, Nucci P, Ratiglia R (2013). In vivo confocal evaluation of the ocular surface morpho-functional unit in dry eye. Optom Vis Sci.

[10] Wakamatsu TH, Sato EA, Matsumoto Y, Ibrahim OM, Dogru M, Kaido M (2010). Conjunctival in vivo confocal scanning laser microscopy in patients with Sjögren syndrome. Invest Ophthalmol Vis Sci.

[11] Villani E, Galimberti D, Viola F, Mapelli C, Ratiglia R (2007). The cornea in Sjogren's syndrome: an in vivo confocal study. Invest Ophthalmol Vis Sci.

[12] Tuisku IS, Konttinen YT, Konttinen LM, Tervo TM (2008). Alterations in corneal sensitivity and nerve morphology in patients with primary Sjögren's syndrome. Exp Eye Res.

[13] Gabbriellini G, Baldini C, Varanini V, Notarstefano C, Pepe P, Fanucci F, Ferro F, Luciano N, Mosca M, Nardi M, Bombardieri S (2015). In vivo confocal scanning laser microscopy in patients with primary Sjögren's syndrome: A monocentric experience. Mod Rheumatol.

[14] Zhang M, Chen J, Luo L, Xiao Q, Sun M, Liu Z (2005). Altered corneal nerves in aqueous tear deficiency viewed by in vivo confocal microscopy. Cornea..

[15] Joana C, Filipe B, Helena C, Diogo H, Sara C, José V, Nuno A (2019). Tear meniscus and corneal sub-basal nerve plexus assessment in primary Sjögren syndrome and Sicca syndrome patients. Cornea..

[16] Shiboski SC, Shiboski CH, Criswell L, Baer A, Challacombe S, Lanfranchi H (2012). American College of Rheumatology classification criteria for Sjogren’s syndrome: a data-driven, expert consensus approach in the Sjogren’s international collaborative clinical Alliance cohort. Arthritis Care Res.

[17] Arita R, Itoh K, Inoue K, Amano S (2008). Noncontact infrared meibography to document age-related changes of the meibomian glands in a normal population. Ophthalmology..

[18] Labbé A, Liang Q, Wang Z, Zhang Y, Xu L, Baudouin C, Sun X (2013). Corneal nerve structure and function in patients with non-sjogren dry eye: clinical correlations. Invest Ophthalmol Vis Sci.

[19] Oliveira-Soto L, Efron N (2001). Morphology of corneal nerves using confocal microscopy. Cornea..

[20] Benítez-Del-Castillo JM, Acosta MC, Wassfi MA, Díaz-Valle D, Gegúndez JA, Fernandez C (2007). Relation between corneal innervation with confocal microscopy and corneal sensitivity with noncontact esthesiometry in patients with dry eye. Invest Ophthalmol Vis Sci.

[21] Kheirkhah A, Dohlman TH, Amparo F, Arnoldner MA, Jamali A, Hamrah P, Dana R (2015). Effects of corneal nerve density on the response to treatment in dry eye disease. Ophthalmology..

[22] Tuominen IS, Konttinen YT, Vesaluoma MH, Moilanen JA, Helinto M, Tervo TM (2003). Corneal innervation and morphology in primary Sjogren's syndrome. Invest Ophthalmol Vis Sci.

[23] Hosal BM, Ornek N, Zilelioglu G, Elhan AH (2005). Morphology of corneal nerves and corneal sensation in dry eye: a preliminary study. Eye..

[24] Chai J, Herrmann DN, Stanton M, Barbano RL, Logigian EL (2005). Painful small-fiber neuropathy in Sjogren sydrome. Neurology..

[25] Kawagashira Y, Koike H, Fujioka Y, Hashimoto R, Tomita M, Morozumi S, Iijima M, Katsuno M, Tanaka F, Sobue G (2012). Differential, size-dependent sensory neuron involvement in the painful and ataxic forms of primary Sjögren's syndrome-associated neuropathy. J Neurol Sci.

[26] Fauchais AL, Richard L, Gondran G, Ghorab K, Palat S, Bezanahary H, Loustaud-Ratti V, Ly K, Jauberteau MO, Vallat JM, Vidal E, Magy L (2011). Small fibre neuropathy in primary Sjögren syndrome. Rev Med Internet.

[27] Wang MTM, Craig JP (2018). Comparative evaluation of clinical methods of tear film stability assessment: A randomized crossover trial. JAMA Ophthalmol.

[28] Mooi JK, Wang MT, Lim J, Muller A, Craig JP (2017). Minimising instilled volume reduces the impact of fluorescein on clinical measurements of tear film stability. Cont Lens Anterior Eye.

[29] Clara LQ, Laura RDV, Syga P (2019). Meibomian gland morphology: the influence of structural variations on gland function and ocular surface parameters. Cornea..

[30] Patel DV, McGhee CNJ (2015). Mapping of the normal human corneal sub-basal nerve plexus by in vivo laser scanning confocal microscopy. Invest Ophthalmol Vis Sci.

[31] Kheirkhah A, Muller R, Mikolajczak J, Ren A, Kadas EM, Zimmermann H, Pruess H, Paul F, Brandt AU, Hamrah P (2015). Comparison of standard versus wide-field composite images of the corneal subbasal layer by in vivo confocal microscopy. Invest Ophthalmol Vis Sci.

[32] Vagenas D, Pritchard N, Edwards K, Shahidi AM, Sampson GP, Russell AW, Malik RA, Efron N (2012). Optimal image sample size for corneal nerve morphometry. Optom Vis Sci.

[33] Allgeier S, Maier S, Mikut R, Peschel S, Reichert KM, Stachs O, Köhler B (2014). Mosaicking the subbasal nerve plexus by guided eye movements. Invest Ophthalmol Vis Sci.

