# Supplementary material for: Corneal nerve structure in patients with primary Sjögren’s syndrome in China
Source: BMC Ophthalmol. 2021 May 12;21:211. doi: 10.1186/s12886-021-01967-7 (PMC8117565; doi:10.1186/s12886-021-01967-7)
Supplement: Supplementary file 1 — Additional file 1: Supplementary Table 1. Statistical Results of Correlations Between Dry Eye Clinical Tests in the SSDE Group. Supplementary Table 2. Statistical Results of Correlations Between Dry Eye Clinical Tests in the NSDE Group. [file 12886_2021_1967_MOESM1_ESM.docx]

Supplementary table 1. Statistical Results of Correlations Between Dry Eye Clinical Tests in the SSDE Group.

| Parameters | age | NIKTMH | NIKBUT | TBUT | Corneal Staining | Schirmer test |
| --- | --- | --- | --- | --- | --- | --- |
| NIKTMH |  |  |  |  |  |  |
| *r* | -0.196 |  |  |  |  |  |
| *P* | 0.383 |  |  |  |  |  |
| NIKBUT |  |  |  |  |  |  |
| *r* | 0.019 | 0.574 |  |  |  |  |
| *P* | 0.932 | 0.007^*^ |  |  |  |  |
| TBUT |  |  |  |  |  |  |
| *r* | -0.647 | 0.047 | 0.042 |  |  |  |
| *P* | 0.001^*^ | 0.840 | 0.857 |  |  |  |
| Corneal Staining | | | | | | |
| *r* | 0.410 | -0.054 | -0.322 | -0.273 |  |  |
| *P* | 0.058 | 0.816 | 0.155 | 0.232 |  |  |
| Schirmer test |  |  |  |  |  |  |
| *r* | -0.300 | -0.232 | -0.418 | 0.049 | 0.134 |  |
| *P* | 0.175 | 0.313 | 0.059 | 0.832 | 0.564 |  |
| Meibomian gland dropout | | | | | | |
| *r* | 0.262 | -0.015 | -0.251 | -0.212 | 0.115 | 0.309 |
| *P* | 0.238 | 0.949 | 0.272 | 0.357 | 0.619 | 0.172 |

^*^ *P* < 0.05

Supplementary table 2. Statistical Results of Correlations Between Dry Eye Clinical Tests in the NSDE Group.

| Parameters | age | NIKTMH | NIKBUT | TBUT | Corneal staining | Schirmer test |
| --- | --- | --- | --- | --- | --- | --- |
| NIKTMH |  |  |  |  |  |  |
| *r* | -0.006 |  |  |  |  |  |
| *P* | 0.980 |  |  |  |  |  |
| NIKBUT |  |  |  |  |  |  |
| *r* | -0.308 | -0.021 |  |  |  |  |
| *P* | 0.187 | 0.930 |  |  |  |  |
| TBUT |  |  |  |  |  |  |
| *r* | -0.136 | -0.039 | 0.567 |  |  |  |
| *P* | 0.567 | 0.873 | 0.011^*^ |  |  |  |
| Corneal Staining | | | | | | |
| *r* | -0.698 | -0.036 | 0.389 | 0.196 |  |  |
| *P* | 0.001^*^ | 0.884 | 0.100 | 0.421 |  |  |
| Schirmer test |  |  |  |  |  |  |
| *r* | 0.327 | 0.287 | -0.585 | -0.458 | -0.508 |  |
| *P* | 0.159 | 0.233 | 0.009^*^ | 0.049^*^ | 0.026^*^ |  |
| Meibomian gland dropout | | | | | | |
| *r* | 0.177 | -0.013 | 0.408 | 0.399 | 0.086 | -0.273 |
| *P* | 0.456 | 0.956 | 0.083 | 0.091 | 0.728 | 0.258 |

^*^ *P* < 0.05
